# 

**Published:** 1866-12

**Authors:** 


LATE.—The Journal will reach its readers next year, owing to unavoidable causes of delay in proof-reading, copy, etc., etc. Accordingly we wish all our friends a "Happy New Year." In future we hope to appear earlier, and be equally entertaini ng and instructive.

### Books and Pamphlets Received.

A Treatise on the Principles and Practice of Medicine; designed for the use of Practitioners and Students of Medicine- By Austin Flint, M. D., Professor of the Principles and Practice of Medicine in the Bellevue Hospital Medical College, and in the Long Island College Hospital; Fellow of the New York Academy of Medicine, etc. Second edition, revised and enlarged. Philadelphia: Henry C. Lea, 1867.

A Treatise on the Principles and Practice of Medicine and Pathology, Diseases of Women and Children, and Medical Surgery. By W. Paine, M. D., Professor of the Principles and Practice of Medicine and Pathology in the Philadelphia University of Medicine and Surgery, etc., etc., and Editor of the University Journal of Medicine and Surgery. Philadelphia: University Pub. Society.

Practical Therapeutics, considered chiefly with reference to Articles of the Materia Medica. By Edward John Waring, F. R. C. S., E. L. S., Surgeon in Her Majesty's Indian Army. From the second London edition. Philadelphia: Lindsay & Blakiston, 1866,

A Handy-Book of Ophthalmic Surgery for the use of Practitioners. By John Z. Laurence, F. R. C. S., M. B. (Univ. London,) Surgeon to the Ophthalmic Hospital, Southwark, etc., etc., and Robert C. Moon, House-Surgeon to the Ophthalmic Hospital, Southwark, with numerous illustrations. London: Robert Hardwicke, 1866.

A Manual of Auscultation and Percussion. By M. Barth and M. Henri Roger. Translated from the sixth French edition. Philadelphia: Lindsay & Blakiston.

The Common Nature of Epidemics and their relations to Climate and Civilization. Also remarks on Contagion and Quarantine, from writings and official reports. By Southwood Smith, M. D., Physician to the London Fever Hospital, etc., etc. Edited by T Baker, Esq., of the Inner Temple, Barrister at Law, etc., etc. Philadelphia: J. B. Lippincott & Co., 1866.

Lessons upon the Diagnosis and Treatment of Surgical Diseases, delivered in the month of August, 1865, by Professor Velpeau, Member de l'Institute et de l'Académie Impériale de Médicine. Translated by W. C. B. Fifield, M. D. Boston: James Campbell, 1866.

Infantile Paralysis, and its Attendant Deformities. By Charles Fayette Taylor, M. D., Resident Surgeon New York Orthopædic Dispensary, etc., etc. Philadelphia: J. B. Lippincott & Co., 1867.

Message and Documents, War Department, 1865-66. Part 3 and 4. Containing Report upon the prevailing diseases of the United States. By Dr. J. H. Baxter.

Insanity in its Medico-Legal Relations. Opinion relative to the Testamentary Capacity of the late James C. Johnston, of Chowan County, North Carolina. By Wm. A. Hammond, M. D.

Address of D. Humphreys Storer, M. D., President of the American Medical Association.

Annual Report of the Surgeon General, United States Army, 1866.

A Classified Priced Catalogue of Medical Books, Surgical Instruments, Apparatus, etc., etc., January, 1867. Published at the office of the Medical and Surgical Reporter, Philadelphia.

Henry C. Lea's New Medical Catalogue.

